# Circulating immune parameters predicting the progression from hospital-acquired pneumonia to septic shock in surgical patients

**DOI:** 10.1186/cc3826

**Published:** 2005-10-12

**Authors:** Vera von Dossow, Koschka Rotard, Uwe Redlich, Ortrud Vargas Hein, Claudia D Spies

**Affiliations:** 1 Resident in Anesthesiology, Department of Anesthesiology and Intensive Care, Charité – Universitaetsmedizin Berlin, Campus Mitte, Germany; 2 Resident in Radiology, Clinic for Radiology and Nuclear Medicine, Charité – Universitaetsmedizin Berlin, Campus Benjamin Franklin, Germany; 3 Resident in Anesthesiology, Department of Anesthesiology, DRK Kliniken Koepenick, Berlin, Germany; 4 Consultant in Anesthesiology, Department of Anesthesiology and Intensive Care, Charité – Universitaetsmedizin Berlin, Campus Mitte, Germany; 5 Professor of Anesthesiology, Head of the Department of Anesthesiology and Intensive Care, Charité – Universitaetsmedizin Berlin, Campus Mitte, Germany

## Abstract

**Introduction:**

Hospital-acquired pneumonia after surgery is one of the major causes of septic shock. The excessive inflammatory response appears to be responsible for the increased susceptibility to infections and subsequent sepsis. The primary aim of this study was to investigate immune parameters at the onset of pneumonia, before the development of subsequent septic shock. The secondary aim was to investigate the usefulness of these immune parameters in predicting progression from hospital-acquired pneumonia to septic shock.

**Methods:**

This propective clinical study included 76 patients with the diagnosis of hospital-acquired pneumonia. Approval was obtained from the local institutional ethics committee and relatives of the patients gave informed consent. Of the 76 patients, 29 subsequently developed septic shock. All patients were included within 4 h of establishing the diagnosis of hospital-acquired pneumonia (first collection of blood samples and the analysis of immune mediators). In addition, we defined early (within 12 h of onset of septic shock) and late (within 72 to 96 h of onset) stages of septic shock for the collection of blood samples and the analysis of immune mediators. The immune parameters tumor necrosis factor-α, IL-1β, IL-6, IL-8 and IL-10 as well as the endothelial leucocyte adhesion molecule were analyzed.

**Results:**

In the pneumonia group with subsequent septic shock, levels of IL-1β, IL-6, IL-8 and IL-10 were significantly increased before the onset of septic shock compared to patients without subsequent septic shock. This progression was best predicted by IL-1β, IL-6, IL-8 and IL-10 (area under the curve ≥ 0.8).

**Conclusion:**

At the onset of hospital-acquired pneumonia, a significant relevant systemic cytokine mediated response had already been initiated. It might, therefore, be possible to identify patients at risk for septic shock with these predictive markers during early pneumonia. In addition, immune modulating therapy might be considered as adjuvant therapy.

## Introduction

Hospital-acquired pneumonia (HAP) is the most common nosocomial infection and its prevalence within the intensive care unit (ICU) setting ranges from 31% to 47% [1-4]. The mortality rate for HAP remains high at 20% to 50% [5-7]. HAP in surgical patients is especially characterized by the high frequency of early onset infections and the high proportion of Gram-negative bacteria and staphylococci isolated [8]. Mortality rates are between 19% and 45% for patients who contract postoperative pneumonia after major surgery [9]. A review by Friedman *et al*. [10] shows that incidents of HAP as a cause of septic shock have increased in the past decades and this has been accompanied by only a limited improvement in survival. In addition, the study of Martin *et al*. [11] shows that HAP as a cause of septic shock was associated with a poor outcome and significantly higher mortality (82%; p < 0.03) compared to wound infections and urinary tract infections.

Several studies indicate that a causal relationship exists between the surgical injury and the predisposition of these patients to develop infectious and septic complications [12,13]. The excessive inflammatory response with alteration of cell-mediated immunity following major surgery appears to be responsible for the increased susceptibility to subsequent infection and sepsis [13]. In particular, the regulation of the inflammatory response in bacterial pneumonia is dependent on complex interactions between alveolar macrophages, polymorphonuclear leukocytes, immune cells and local production of both pro- and anti-inflammatory cytokines as well as vascular adhesion molecules [14-16]. The cytokines secreted by phagocytes in response to infection include tumor necrosis factor (TNF)-α, IL-1β, IL-6, IL-8 as well as IL-10 [14]. Interleukins are increasingly recognized as early mediators of the host inflammatory response to a variety of infectious agents. On one hand, cytokines can leak from the inflammatory sites in the lung as the normal compartmentalization of inflammation is lost during severe local infection [17,18]. Alternatively, cytokines may be produced in the systemic compartment in response to bacterial products, such as endotoxin, that leak from the lungs into the circulation [19-21]. Parsons *et al*. [22] have provided the strongest evidence to date that IL-6, IL-8 and IL-10 are useful circulating markers for the intensitiy of the inflammatory response in the lungs and the prognosis of patients at the onset of lung injury. High elevated plasma levels of IL-6 and IL-8 have been associated with higher mortality rates [23-25].

To the best of our knowledge, no other study to date has investigated the systemic progression of HAP to septic shock with respect to the pattern of circulating cytokines in surgical patients.

The primary aim of this study was to investigate whether patients, within four hours of a HAP diagnosis, differed in their pro- and anti-inflammatory cytokine and adhesion molecule patterns before the development of septic shock. The secondary aim was to evaluate whether any of these markers had a predictive value for the progression of HAP to septic shock.

## Materials and methods

This study was approved for an operative ICU. After receiving both the approval of the institutional ethics commitee and written informed consent from patients relatives or legal representatives, 76 patients were included. All patients were surgical patients (major abdominal, neurosurgical, trauma). The patients were allocated to two groups: HAP without septic shock and HAP with subsequent septic shock. HAP was diagnosed according to the criteria of the American Thoracic Society 1996 [2]. Patients were included within 4 h after the onset of HAP. Exclusion criteria were patients younger than 18 years old, acute myocardial ischemia, any corticosteroid therapy or chemotherapy, acute respiratory distress syndrome (ARDS), acute lung injury and heart insufficiency. A diagnosis of pneumonia was made if systemic signs of infection were present, new or worsening infiltrates were seen on the chest X-ray, and new onset of purulent sputum or a change of sputum with bacteriologic evidence in the endotracheal aspirate was found [26,27]. Subsequent septic shock criteria were defined as outlined in the Consensus Conference 1992 [28]. In particular, we defined early (within 12 h of onset) and late (within 72 to 96 h of onset) stages of septic shock.

The collection of blood samples for the analysis of immune mediators, were drawn in all patients (with and without subsequent septic shock) at the time of HAP diagnosis within the first 4 h after onset. In patients with HAP and subsequent septic shock, blood samples were obtained at the early stage (within 12 h of onset) as well the late stage (within 72 to 96 h of onset) of subsequent septic shock.

All blood samples were collected in sterile tubes and centrifuged; the supernatants were stored in liquid nitrogen at -70°C. All mediators were analyzed at 23°C using a sandwich enzyme-linked immunosorbent assay (Quantikine™ Immunoassay Kit, R&D Systems, Minneapolis, MN, USA). Detection limits were: IL-1β, 0.1 pg/ml (intra- and interassay variation coefficients 3.0% and 12.5%, respectively); IL-6, 3 pg/ml (4.6%, 12.1%); IL-8, 8 pg/ml (5%, 11.1%); IL-10, 5 pg/ml (3.0%, 7.0%); TNF-α, 4.4 pg/ml (4.6%, 5.8%); E-selectin, 2 ng/ml (3.2%, 6.4%).

Routine laboratory parameters, including leucocytes, C-reactive protein (CRP), lactate and platelets, were determined two times a day. All patients were mechanically ventilated and received a continuous analgosedation with either propofol/fentanyl or midazolam/fentanyl. Basic patient characteristics, the microbiological etiology pneumonia, the Acute Physiology and Chronic Health Evaluation (APACHE) III score [29] and the Multiple Organ Failure (MOF) score [30] were documented. The researchers who performed the laboratory analyses were blinded to data collection, diagnosis of pneumonia and ICU outcome. Furthermore, the diagnosis of HAP made by clinicians on the ICU was seen and confirmed by two blinded researchers.

A radial artery catheter and a central-venous catheter were inserted as routine monitoring in all patients. A pulmonary artery catheter was inserted as routine cardiovascular monitoring for the 29 patients with subsequent septic shock. Arterial and mixed-venous blood gases were performed in all patients with septic shock to determine oxygen-transport related variables, in particular oxygen delivery and oxygen consumption via standard formulae. Volume resuscitation (crystalloids, colloids and blood transfusions) was performed to achieve an optimal left arterial pressure, which was estimated by the pulmonary capillary wedge pressure reaching the plateau value for left ventricular stroke work. If the cardiac index was <2.5 l/minute/m^2^, 3 to 10 ug/kg/minute dobutamine was administered to maintain the cardiac index between 3.0 and 3.5 l/minute/m^2^. If mean arterial pressure was below the level of 70 mmHg, norepinephrine was administered to obtain a mean arterial pressure between 70 and 90 mmHg [31]. Steroids were given in patients at the time of septic shock according to additive therapy, especially in patients who exhibited a poor response to the primary vasopressor agent [31].

### Statistics

All data are expressed as median and 25/75 percentile. Statistical analysis between groups (HAP patients with and without subsequent septic shock) was performed using the Mann-Withney U test (intergroup analysis). The receiver operating curve was plotted to provide a graphical presentation of the relationship between sensitivity and specificity of the mediators covering all possible diagnostic cutoff levels. The area below the receiver operating curve (AUC) represents the probability of septic shock developing in a patient with pneumonia [32]. Statistical analysis of the pneumonia patients with subsequent septic shock (intragroup analysis) was performed using the Friedman test to show significant differences between pneumonia and early and late septic shock. When the global test revealed a significant difference, the Wilcoxon matched-pairs signed-rank test was then used to decide whether or not pneumonia and early and late septic shock differed locally. The Chi-square test was used to test statistical differences between dichotomous variables. A p < 0.05 was considered significant.

## Results

Out of a total of 76 patients with HAP, 29 patients developed subsequent septic shock. Basic patient characteristics as well as the etiology of pneumonia (Gram-positive/Gram-negative) did not differ significantly between the two groups (Table 1). In the pneumonia group without septic shock, Gram-positive species were isolated from 18 patients (*Staphylococcus aureus*, *Enterococcus faecium*, *Enterococcus faecalis*), whereas Gram-negative species were isolated from 21 patients (*Pseudomonas aeruginosa*, *Proteus mirabilis*, *Klebsiella pneumoniae*, *Enterobacter cloacae*). In the pneumonia group with subsequent shock, eight patients had Gram-positive pulmonary infection (*Staphylococcus aureus*, *Enterococcus faecium*, *Enterococcus faecalis*), whereas Gram-negative species were isolated from 15 patients (*Pseudomonas aeruginosa*, *Proteus mirabilis*, *Klebsiella pneumoniae*, *Enterobacter cloacae*).

At the 'diagnosis of pneumonia', the clinical and laboratory findings did not significantly differ between the groups (Table 2). The time from admission to the ICU to the time of diagnosis of pneumonia did not differ between the groups (p < 0.37; Table 2). In the pneumonia group with subsequent septic shock, the time from diagnosis of pneumonia to the onset of early septic shock was 75 h (range 30 to 85 h), from early septic shock to late septic shock 78 h (range 17 to 103 h). None of the patients of the septic shock group had septic shock at the time of diagnosis of pneumonia. The progression from pneumonia to septic shock showed a significant increase in APACHE III and MOF scores as well in levels of leucocytes, although there was no significant increase in the levels of C-reactive protein, lactate and platelets (Table 3).

### Immune modulating mediators and clinical parameters at the onset of pneumonia and during subsequent septic shock

**Figure 1:**
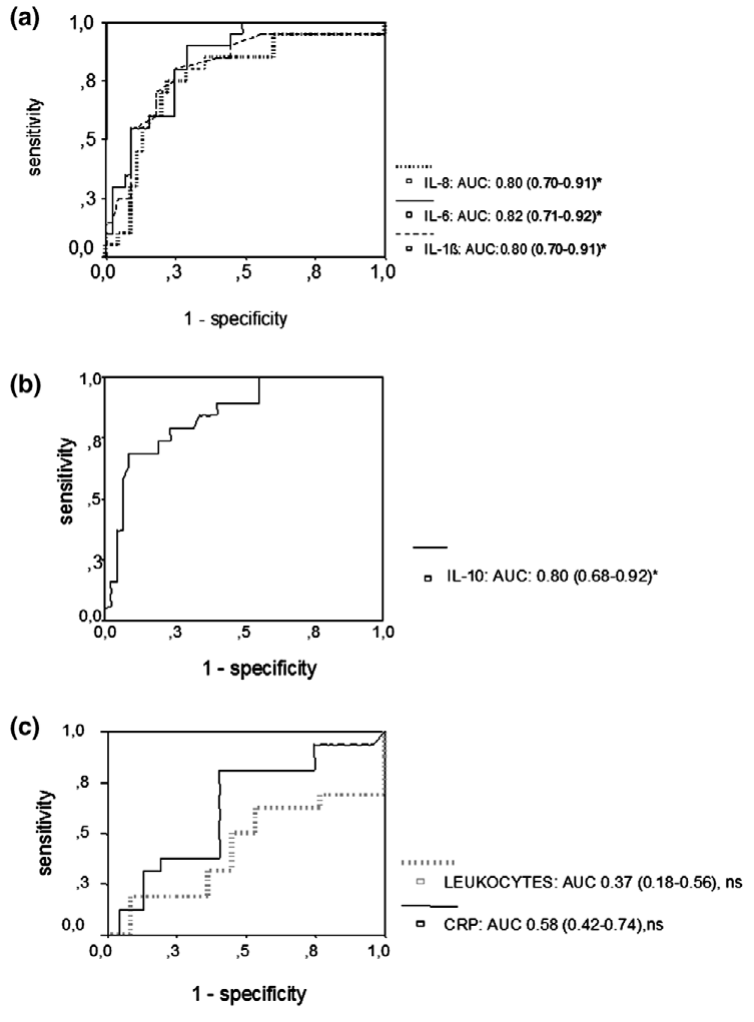
Predictive value of immune modulating parameters and conventional laboratory parameters at hospital-acquired pneumonia diagnosis. **(a) **Area under the receiver operating curve (AUC) for IL-6, IL-8 and IL-1β (*p < 0.05). **(b) **AUC for IL-10 (*p < 0.05). **(c) **AUC for C-reactive protein (CRP). Ns, not significant.

At the 'diagnosis of pneumonia', TNF-α, IL-1β, IL-6, IL-8, IL-10 and E-selectin were significantly increased in those patients who had subsequent septic shock, compared to patients with pneumonia without subsequent septic shock (Table 4). The AUC of IL-1β, IL-6, IL-8 and IL-10 ranged from 0.80 to 0.82 (Fig. 1a,b). In addition, routine laboratory parameters, such as levels of lactate, leucocytes and C-reactive protein as well as APACHE III and MOF scores, did not differ between the groups. The AUC of leukocytes and C-reactive protein ranged from 0.34 to 0.58 (Fig. 1c).

TNF-α and E-selectin increased significantly in 'early' septic shock. From 'early' to 'late' septic shock, significant decreases were observed in TNF-α, IL-1β, IL-6, and E-selectin (Table 3).

### Hemodynamic and oxygen-related parameters in septic shock patients

None of the patients in both the non-septic and the septic shock group had pre-existing ARDS or fulfilled the criteria of ARDS at the diagnosis of pneumonia. None of the patients of either group had bilateral infiltrates in the chest X-ray as a radiomorphological correlate for the diagnosis of ARDS. The heart rate of patients with septic shock was significantly higher in the late phase of septic shock. In addition, oxygen consumption was significantly higher in early septic shock.

### ICU stay and outcome in patients without and with septic shock

ICU stay did not differ between both groups. In contrast, the survival rate was significantly higher in patients without septic shock (Table 5). For 12 patients (5 patients without subsequent septic shock and 7 patients with subsequent septic shock), initial inappropriate antibiotic therapy was changed immediately according to the specific bacterial strains isolated. No significant differences in inflammatory parameters were found in these patients compared to patients who received initial adequate therapy.

## Discussion

The most important result of this study was the detection of an already increased immune response with respect to circulating cytokines at the onset of HAP in all patients with subsequent septic shock compared to those without subsequent septic shock. In particular, IL-1β, IL-6, IL-8 and IL-10 were most predictive for the progression of septic shock (area under the curve ≥ 0.80). Furthermore, in our study, laboratory markers were not predictive for the progression of HAP to septic shock, which is in accordance with other clinical studies [11].

To the best of our knowledge, no other study to date has investigated the systemic progression of HAP to septic shock in surgical patients with respect to immune modulating cytokines, adhesion molecules, their systemic release and their possible predictive value.

In our study, increased serum levels of IL-1β, IL-6, IL-8 and IL-10 were found at the onset of HAP and had a predictive value (area under the curve ≥ 0.80) for the progression to septic shock. An increase in levels of IL-6 and IL-10 has been reported after different types of surgery [33,34]. Furthermore, different early proinflammatory cytokine responses, as well as exaggerated increases in IL-10, are associated with later onset of infections [35,34]. Brede *et al*. [35] demonstrated an immediate increase in plasma TNF-α levels in peritonitis patients, which was predictive for the development of subsequent septic shock. None of the patients had septic shock at the diagnosis of peritonitis, which is in accordance with our findings. In addition, Sander *et al*. [36] found decreased IL-6/IL-10 levels in patients immediately after surgery of the upper gastrointestinal tract, which was associated with an increased risk of postoperative infections. Spies *et al*. [37] reported an immediate increased IL-10 response, which was associated with the later onset of postoperative infections. Even if the above mentioned studies are not fully comparable to our study, the postoperative and early immediate increase of cytokines, especially IL-10, IL-1β, IL-8 and IL-6, might be explained as an exaggerated and imbalanced pro- and anti-inflammatory immune response after surgery.

In general, a clinical complication of HAP is the dissemination of bacteria from the pulmonary airspace into the bloodstream, resulting in bacteremia concurrent with the localized infection [38,39]. In addition to direct bacterial phagocytosis, alveolar macrophages secrete a variety of cytokines and chemokines capable of activating blood neutrophils and monocytes in the pulmonary microenvironment [38]. Furthermore, inability to clear bacteria from the bloodstream can lead to a high exposure to endotoxin and subsequent septic shock [39,40]. The cytokines secreted by phagocytes in response to infection include TNF-α, IL-1β, IL-6, IL-8 and IL-10 [14]. In accordance with our findings, Bonten *et al*. [23] showed that ventilator-associated pneumonia in patients with severe sepsis and septic shock was accompanied by increased levels of IL-6 and IL-8 at the time of diagnosis, and even two days after diagnosis, compared to control patients without ventilator-associated pneumonia. Furthermore, Meduri *et al*. [41] found a persistent elevation of the cytokines TNF-α, IL-1β, IL-6 and IL-10 after the diagnosis of adult respiratory distress syndrome, which predicted a poor outcome and severe sepsis and septic shock. In contrast, Friedland *et al*. [10] found that circulating levels of IL-6, IL-8 and IL-1β poorly correlated with the clinical severity of illness of ICU patients and only the presence of TNF-α in plasma was an independent predictor of mortality. However, it is difficult to make comparisons to the study of Friedland *et al*. [10] because of its heterogenous patient population (surgical and medical emergencies). In addition no differentiation with respect to the infectious focus was made in the study of Friedland et al. [10].

Experimental investigations documented a leakage of pro-inflammatory parameters from the infected lung [17], which caused increased systemic levels of pro- and anti-inflammatory cytokines in the circulation [17,18]. In an experimental rabbit model, Kurahashi *et al*. [18] studied the pathogenesis of septic shock in *Pseudomonas aeruginosa *pneumonia with lung instillation of a cytotoxic PA 103, which caused a significant bacterial-induced alveolar epithelial injury and a progressive increase in the circulating pro-inflammatory cytokines TNF-α and IL-8. Pretreatment with systemic administration of anti-TNF-α serum or rh-IL-10 blocked the increase of pro-inflammatory mediators in the circulation and prevented hypotension and decreased cardiac output. Our impression is that this experimental setting indicates that systemic inflammation is already present at an early stage of infection and that specific immune parameters are related to the focus of infection and might thus be used to predict a worse outcome.

In our study, the outcome was significantly different between groups. Of 29 patients with subsequent septic shock, 13 (44.8%) died. This is in accordance with previous studies [3,23,42]. In the study of Bonten and coworkers [23], the mortality rate for ventilator-associated pneumonia patients with subsequent septic shock was 60%. Ibrahim *et al*. [3] studied 301 patients receiving mechanical ventilation. The mortality rate of patients who developed ventilator-associated pneumonia (45.5%) was significantly greater than the mortality rate of patients without ventilator-aquired pneumonia (32.2%, p < 0.004). Almiralli *et al*. [42] studied a total of 127 patients with community-aquired pneumonia (45.7%) and HAP (54.3%). Of the patients with HAP, 18.8% developed subsequent septic shock, which was associated with an increased mortality (66%). In addition, other predictive variables, such as a Simplified Acute Physiology II Score >12, mechanical ventilation and advanced age (>70 years) were associated with a 99% probability of a fatal outcome [42]. In contrast to the studies mentioned above, the APACHE III and MOF scores in our study were not predictive at the onset of HAP for the progression to subsequent septic shock. It is difficult, however, to compare these studies to our study because of their different patient populations and study designs.

A relationship between elevated levels of cytokines and both a clinical condition of severe sepsis or septic shock and mortality have been demonstrated repeatedly [20,23]. It has been suggested that systemically stimulated immune parameters may predict a worse outcome [20,23], which is in accordance with our study as progression from HAP to septic shock was associated with an early increase in the above mentioned cytokines prior to the development of septic shock. The subsequent decrease of nearly all immune modulating parameters during late septic shock may be considered as an immune breakdown, indicating an immune paralytic effect. This has been shown already in patients with peritonitis and subsequent septic shock [35]. The significant increase in MOF and APACHE III scores during early and late septic shock reflect the severity of septic shock and the development of multiple organ failure [43].

### Limitations of the study

In this study, we did not perform a bronchial lavage procedure or determine cytokine levels in the bronchial lavage. This might be a limitation of this study, but direct examination of reliable respiratory tract samples cannot be relied upon solely [44]. Even if previous studies demonstrated elevated levels of IL-8 and IL-10 in the bronchial lavage of injured patients at the time of admission, which was associated with subsequent nosocomial pneumonia, this was not predictive for the development of subsequent septic shock [45]. The exact timing of HAP diagnosis was taken into consideration according to the precisely defined criteria of the American Thoracic Society 1996 [2] and is essential for timely administration of appropriate antibitiotic therapy [28,44]. The sepsis definition is much more difficult and was performed according to the American College of Chest Physicians and Society of Critical Care Medicine 1992 [28]. Whereas it cannot be ruled out that 12 patients received inappropriate initial antibiotic therapy, no significant differences were observed for them with respect to outcome and inflammatory parameters.

## Conclusion

In this study, a significant and clinically relevant systemic cytokine mediated response had already been initiated at the onset of HAP. This prestimulated response had a better predictive capacity for subsequent septic shock than conventional laboratory values. In HAP patients with subsequent septic shock, IL-1β, IL-6, IL-8 and IL-10 were superior predictive markers than TNF-α, E-selectin and conventional laboratory values and scores at the time of pneumonia diagnosis. It is possible, therefore, to identify patients at risk of septic shock in early pneumonia with these predictive markers.

## Key messages

• 	At the time of HAP diagnosis, TNF-α, IL-1β, IL-6, IL-8, IL-10 and E-selectin were significantly increased in pneumonia patients with subsequent septic shock compared to patients without subsequent septic shock.

• 	In particular, IL-1β, IL-6, IL-8, and IL-10 were most predictive for the progression of septic shock (area under the curve ≥ 0.8).

• 	Conventional laboratory markers as well as APACHE III and MOF scores were not predictive for subsequent septic shock.

• 	At the time of HAP diagnosis, a significant and clinically systemic cytokine mediated response had already been initiated and had predictive capacity for subsequent septic shock.

• 	In the clinical context, it might be possible to identify patients at risk of septic shock in early HAP with predictive markers.

## Abbreviations

ARDS = acute respiratory distress syndrome; AUC = area under curve; CRP = C-reactive protein; HAP = hospital-acquired pneumonia; ICU = intensive care unit; IL = interleukin; TNF = tumor necrosis factor.

## Competing interests

The authors declare that they have no competing interests.

## Authors' contributions

VvD participated in the study design, interpretation of the results, and was involved in writing and revising the manuscript critically for important intellectual content. KR was involved in acquisition of data. UR was involved in the statistical analysis of data and helped to draft the manuscript. OVH participated in the study design and helped in drafting the manuscript. All above mentioned authors read and approved the final manuscript. CS conceived the study, participated in the study design, interpretation of the results, and in writing of the article, revised the manuscript critically for important intellectual content, and gave final approval of the version to be published.

## Figures and Tables

**Table 1:** Basic patient characteristics and etiology of pneumonia at the time of admission to the intensive care unit

Characteristics and etiology (n = 76)	Pneumonia without subsequent septic shock (n = 47)^a^	Pneumonia with subsequent septic shock (n = 29)^a^	p value
Age (years)	49 (36–58)	54 (47–69)	0.18
BSA (m^2^)	1.9 (1.8–2.0)	1.9 (1.8–2.0)	0.64
APACHE III	39 (31–50)	38 (32–53)	0.58
MOF score	3.0 (1.0–4.0)	4.5 (2.7–6.0)	0.09
Infections Gram+/Gram-	18/21	8/15	= 0.99

^a^Data are expressed as median (25/75 percentile). APACHE III, Acute Physiology and Chronic Health Evaluation III score at the time of admission to the intensive care unit; BSA, body surface area; Gram+, Gram-positive; Gram-, Gram-negative; MOF, Multiple Organ Failure score.

**Table 2:** Scoring systems and laboratory findings at the time of hospital-acquired pneumonia diagnosis

Clinical and laboratory findings (n = 76)	Pneumonia without subsequent septic shock (n = 47)^a^	Pneumonia with subsequent septic shock (n = 29)^a^	p value
Time from admission to onset of pneumonia (h)	33.0 (4.0–87.0)	42.0 (22.0–78.0)	0.37
CRP (U/l)	60.0 (33–101)	80.0 (71.0–135.0)	0.13
Leukocytes (G/l)	12.7 (10.1–15.0)	12.6 (4.4–13.3)	0.18
Platelets (G/l)	175.0 (135.0–325.0)	193.0 (79.0–435.0)	0.51
Lactate (mmol/l)	1.3 (1.1–1.7)	1.5 (1.2–2.4)	0.05
PaO_2 _ratio/F_I_O_2_	350 (300–375)	350 (310–385)	0.24

^a^Data are expressed as median (25/75 percentile). CRP, C-reactive protein; F_I_O_2_, inspired oxygen concentration; G/l, cells × 10^9 ^per liter; PaO_2_, arterial oxygen pressure.

**Table 3:** Scores, laboratory findings and immune modulating parameters in hospital-acquired pneumonia patients with subsequent septic shock

Clinical and laboratory findings (n = 29)	Pneumonia with subsequent septic shock (I)^a^	Early septic shock (II)^a^	Late septic shock (III)^a^	p value^b^
MOF	4 (3–6)	6 (5–8)	7 (4–9)	≤ 0.01 (I-II, I-III)
APACHE III	38 (32–52)	56 (39–75)	66 (45–88)	≤ 0.01 (I-II, I-III)
CRP (U/l)	80 (71–135)	131 (76–260)	103 (44–225)	0.60
Leucocytes (G/l)	12.6 (4.4–13.3)	18.9 (14.9–26.5)	19.8. (14.0–29.4)	≤ 0.01 (I-II, I-III)
TNF-α (pg/ml)	12.0 (8.0–15.2)	21.0 (9.8–55)	12.0 (9.7–16.7)	≤ 0.01 (I-II, II-III)
IL-1β (pg/ml)	1.9 (1.4–2.0)	2.0 (1.7–2.85)	1.5 (1.2–1.9)	≤ 0.01 (II-III)
IL-6 (pg/ml)	367 (166–678)	773 (229–1,370)	253 (98–1,370)	0.01 (II-III)
IL-8 (pg/ml)	187 (106–410)	271 (108–638)	215 (98–1,370)	0.17
IL-10 (pg/ml)	47 (21–144)	45 (31–120)	26 (48–92)	0.16
E-selectin (ng/ml)	71 (42–115)	134 (78–184)	74 (48–92)	≤ 0.01 (I-II, II-III)

^a^Data are expressed as median (25/75 percentile). ^b^I-II, II-III and I-III: significant difference between measurement I and II, I and III, and II and III (Wilcoxon matched-pairs signed-rank test) if globally found by Friedman test. APACHE III, Acute and Chronic Health Evaluation III score; CRP, C-reactive protein; MOF, Multiple Organ Failure score; TNF, tumor necrosis factor.

**Table 4:** Immunmodulatory parameters at the time of hospital-acquired pneumonia diagnosis

Immunmodulatory parameters (n = 76)	Pneumonia without subsequent septic shock (n = 47)^a^	Pneumonia with subsequent septic shock (n = 29)^a^	p value
TNF-α (pg/ml)	7.0 (6.0–9.0)	12.0 (8.0–15.0)	≤ 0.01
IL-1β (pg/ml)	1.2 (1.0–1.4)	1.85 (1.3–2.0)	≤ 0.01
IL-6 (pg/ml)	64.0 (29.0–155.0)	367.0 (166.5–678.1)	≤ 0.01
IL-8 (pg/ml)	73.0 (52.0–103.0)	187.0 (106.1–410.0)	≤ 0.01
IL-10 (pg/ml)	15.0 (8.0–24.0)	47.0 (21.7–144.0)	≤ 0.01
E-selectin (ng/ml)	34.0 (24.0–56.0)	71.0 (42.5–115.0)	≤ 0.01

^a^Data are expressed as median (25/75 percentile). p < 0.05. E-selectin, endothelial leukocyte adhesion molecule; TNF, tumor necrosis factor alpha.

**Table 5:** Intensive care unit outcome

Outcome (n = 76)	Pneumonia without subsequent septic shock (n = 47)^a^	Pneumonia with subsequent septic shock (n = 29)^a^	p value
MOF (worst score during ICU stay)	4 (3–5)	8 (6–9)	≤ 0.01
ICU stay (days)	12 (8–18)	19 (9–30)	0.06
Survivor/non-survivor^b^	67/0	16/13	≤ 0.01

^a^Data are expressed as median (25/75 percentile). ^b^Survivor/non-survivor analyzed by X^2^-test. ICU, intensive care unit; MOF, Multiple Organ Failure score.
